# Progenitor Cells for Arterial Repair: Incremental Advancements towards Therapeutic Reality

**DOI:** 10.1155/2017/8270498

**Published:** 2017-01-23

**Authors:** Trevor Simard, Richard G. Jung, Pouya Motazedian, Pietro Di Santo, F. Daniel Ramirez, Juan J. Russo, Alisha Labinaz, Altayyeb Yousef, Brijesh Anantharam, Ali Pourdjabbar, Benjamin Hibbert

**Affiliations:** ^1^CAPITAL Research Group, Division of Cardiology, University of Ottawa Heart Institute, Ottawa, ON, Canada; ^2^Department of Cellular and Molecular Medicine, University of Ottawa, Ottawa, ON, Canada; ^3^Interventional Cardiology, University of California, San Diego, La Jolla, CA, USA

## Abstract

Coronary revascularization remains the standard treatment for obstructive coronary artery disease and can be accomplished by either percutaneous coronary intervention (PCI) or coronary artery bypass graft surgery. Considerable advances have rendered PCI the most common form of revascularization and improved clinical outcomes. However, numerous challenges to modern PCI remain, namely, in-stent restenosis and stent thrombosis, underscoring the importance of understanding the vessel wall response to injury to identify targets for intervention. Among recent promising discoveries, endothelial progenitor cells (EPCs) have garnered considerable interest given an increasing appreciation of their role in vascular homeostasis and their ability to promote vascular repair after stent placement. Circulating EPC numbers have been inversely correlated with cardiovascular risk, while administration of EPCs in humans has demonstrated improved clinical outcomes. Despite these encouraging results, however, advancing EPCs as a therapeutic modality has been hampered by a fundamental roadblock: what constitutes an EPC? We review current definitions and sources of EPCs as well as the proposed mechanisms of EPC-mediated vascular repair. Additionally, we discuss the current state of EPCs as therapeutic agents, focusing on endogenous augmentation and transplantation.

## 1. Introduction 

Coronary artery disease (CAD) remains a leading cause of morbidity and mortality [1]. Coronary revascularization (restoring blood flow to the myocardium) remains the standard treatment for obstructive CAD and can be accomplished by either percutaneous coronary intervention (PCI) or coronary artery bypass graft (CABG) surgery. PCI has become the most frequently performed means of revascularization, aided by considerable advances in the field. What started as simple balloon angioplasty has now evolved through many generations of vascular scaffolds, from bare-metal stents (BMSs) to first and then second generation drug-eluting stents (DESs) [2–5]. While early technologies in PCI were fraught with high rates of complications including abrupt vessel closure, subsequent advances have mitigated much of this risk. Nevertheless, numerous challenges remain, including in-stent restenosis (ISR) and stent thrombosis (ST), collectively occurring in 5–8% of cases per year [6, 7]. Moreover, our understanding of the vessel wall's response to injury continues to evolve offering further opportunities for therapeutic intervention as our understanding of the impact that progenitor populations play has crystallized. Over the last decade, characterizing progenitor cell populations, understanding their role in vascular homeostasis, and harnessing their therapeutic potential by modifying them at a biological level remain areas of intense investigation.

The vessel wall's response to injury (e.g., plaque disruption and/or stent deployment) depends on many interdependent factors, including the insult itself in combination with the dynamic nature of the vessel wall-stent interface (including the cellular composition of the vessel wall and stent material and dimensions), vascular tone, and circulating signals. Indeed, PCI itself prompts endothelial denudation and disruption of vascular homeostasis given the high pressure balloon inflation and subsequent force transmitted by stent struts to the endothelial layer extending damage through the underlying medial and adventitial layers [8]. In response to injury, the endothelium secretes a variety of molecules that influence vascular tone, inflammatory cell invasion, thrombus formation, and smooth muscle cell proliferation [9]. Indeed, stent-induced endothelial injury leads to platelet activation and thrombus propagation which alone can result in stent thrombosis [10]. As well, injury mediated inflammation is driven by activation of hypoxia-induced factor (HIF), which has a downstream effect on a number of factors including stromal cell derived factor-1 (SDF-1), angiopoietin, interleukin-8 (IL-8), vascular endothelial growth factor (VEGF), and c-Kit ligand to name a few [11]. Collectively, this activation stimulates the influx of inflammatory cells (neutrophils, monocytes, and macrophages) into the vascular wall, while also stimulating smooth muscle cells (SMCs) and myofibroblasts leading to neointima (NI) formation and subsequent ISR [12]. Paradoxically, this process, including VEGF secretion in particular, provides a stimulus for mobilizing endothelial progenitor cells (EPCs) to the site of injury and mediating vascular repair [13], the mechanisms of which are reviewed in detail below.

EPCs represent an attractive therapeutic option given their purported ability to promote vascular repair after stent placement. Accordingly, an inverse correlation between circulating EPC numbers and both atherosclerotic burden and probability of future cardiovascular risk has been noted [14, 15]. Furthermore, patients with ISR also have lower numbers of circulating EPCs and impaired EPC function [16, 17]. Given the potential benefits of EPC activity at sites of vascular injury, means of augmenting their number and/or function has been pursued and bolstered by clinical and animal studies suggesting improved outcomes following administration of EPCs [18, 19]. Furthermore, evidence suggests that EPCs are mobilized in patients with acute coronary syndromes, suggesting the presence of an endogenous mechanism that, if harnessed, could potentially improve outcomes [20, 21]. This work has stimulated interest in the importance of EPC signaling, number, and function in modulating vascular homeostasis with the potential for therapeutic benefit by capitalizing on EPC biology, particularly in the setting of PCI [22]. Herein, we review the biology of EPCs and their role as marker, modulator, and therapeutic agent for vascular repair.

## 2. Endothelial Progenitor Cells

**Figure 1:**
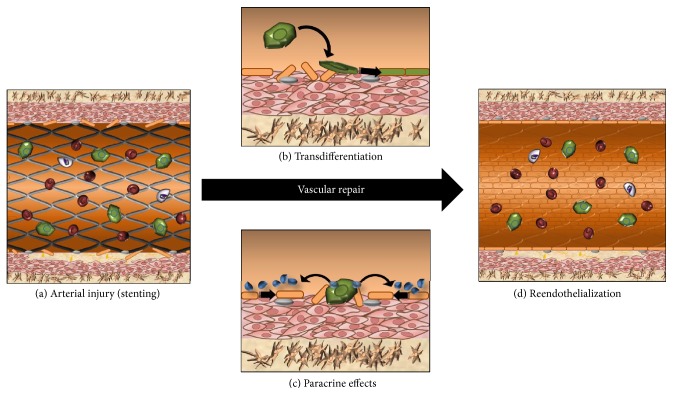
Mechanisms of EPC-mediated vascular repair: (a) coronary stenting resulting in arterial injury. Placement of coronary stent to treat obstructive coronary artery disease (CAD) with plaque being pushed into vessel wall and denudation of endothelial cells (ECs) causing vascular injury. Circulating EPCs then home to the site of vascular injury and mediate repair via either transdifferentiation or paracrine effects. (b) Transdifferentiation of EPCs: EPC transdifferentiates into ECs (black arrows) to repair denuded endothelium. (c) Paracrine effects of EPCs: EPCs secrete numerous chemokines (blue) which stimulate surrounding cells to proliferate and migrate towards site of injury to regenerate the EC layer (black arrows). (d) Reendothelialization: endothelial layer is restored with coronary stent underlying regenerated EC layer. Adventitia (brown), media (pink), endothelial cells (orange), EPCs (green), erythrocytes (red), and leukocytes (white).

Vascular progenitor cells were first observed in animal models when implanting Dacron grafts or silastic tubes [23–29]. These experiments first indicated the existence of a “pseudointima” composed primarily of endothelial cells and vascular smooth muscle cells, generating interest as to the origin of these cells [30]. This origin was finally discovered in 1997, when Asahara isolated EPCs from peripheral blood, describing them as CD34+ cells circulating in the human vasculature which were subsequently implicated in vascular homeostasis and endothelial repair [5, 31–34]. Subsequent studies described the incorporation of bone marrow-derived EPCs into blood vessels following hind limb ischemia [35] and myocardial infarction [36] as well as at wound [37] and tumour [38] sites. EPCs are believed to promote vascular repair by homing to sites of vascular injury where they act via [1] paracrine signaling to neighbouring cells (now believed to represent the predominant mode of action) and [2] transdifferentiating to mature endothelial cells and undergoing angiogenesis to form new blood vessels [5, 39] (Figure 1). In this way, EPCs are believed to hold therapeutic promise, though harnessing this potential has proven challenging.

### 2.1. The Evolving Definition of EPCs

**Figure 2:**
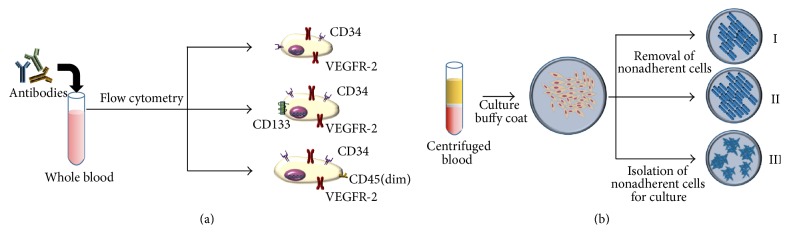
Definition of endothelial progenitor cells (EPCs). (a) Flow cytometry: analysis of whole blood samples by flow cytometry utilizing various combinations of antibodies tagged with fluorophores raised against surface antigens such as CD34+/VEGFR-2+, CD34+/VEGFR-2+/CD133+, or CD34+/VEGFR-2+/CD45(dim) to isolate populations. (b) Culture assay: obtained via Ficoll-based centrifugation of whole blood to isolate and culture the buffy-coat layer composed of peripheral blood mononuclear cells (PBMCs). I and II use adherent cells after removal of nonadherent cells. I, EPC culture on fibronectin plate; II, culturing these cells on collagen plate forming endothelial colony forming cells; III, isolation of nonadherent cells for culture to generate colony forming units (CFUs).

Since their discovery, the precise definition of EPCs remains the subject of considerable debate and represents a major hindrance in understanding the biology and application of these unique cells. This is believed to be a driving force behind conflicting results noted in both preclinical and clinical trials, where differing methodologies in cell sources, cell purification, detection methods, and animal models or assays have been published [40]. Thus far, EPCs have been identified through two main methods: (1) cell-surface markers and (2) colony forming units (CFUs) and cultured/circulated angiogenic cells (CACs) (Figure 2). However, while these methods allow for the physical identification of EPCs, they are unable to characterize the in vitro activity of these cells nor the phenotype they express in physiologic conditions. We herein briefly discuss two common methods used to define EPCs.

#### 2.1.1. Identification by Cell-Surface Markers

EPCs can be classified based on the expression of specific cell-surface antigens via flow cytometry (Table 1). Mature endothelial cells (ECs) express multiple surface antigens including CD34, VE-cadherin, von Willebrand factor (vWF), kinase insert domain-containing receptor (KDR) or VEGFR2, and E-selectin [41]. However, surface antigen expression varies between mature ECs depending on the specific vessel, organ, and activation state of the endothelium. To distinguish between EPCs and mature ECs shed after vascular injury, CD133 (AC133) was utilized, given that it is typically lost during maturation of EPCs into mature ECs. Surface markers CD34, VEGFR2, and CD133 have been widely accepted by researchers for defining EPCs, though many studies typically employ two of the three receptors [40, 41]. Other studies of EPCs have employed CD45dim/CD34/CD133/CD117, with CD117 playing a role in mobilization of hematopoietic stem cells [32, 42, 43]. In 2007, Case et al. isolated EPCs from adult peripheral blood by targeting CD34+ VEGFR2+ and CD133+ cells which did not contribute to the formation of mature endothelial cells in vitro, further challenging the existing models of progenitor cells [44]. Despite controversy, cell-surface marker-based definitions of EPCs (namely, CD34+/KDR+) have been demonstrated to be predictive of cardiovascular outcomes in patients, lending support to this approach [15]. Further work, including in vivo and in vitro assessment of endothelial differentiation capacity, is required to better define this subpopulation of progenitors and ensure future studies and therapies focus on a homogeneous population.

#### 2.1.2. Identification by Culture-Based Techniques

EPCs can also be isolated from peripheral blood by plating these cells in various cell culture assays and assessing their colony forming ability (Figure 2). Asahara et al. first identified these CD34+ EPCs based on cluster-forming cells [31]. Colony counts were used as a predictor of the number of circulating progenitors while the spindle-shaped cells surrounding each colony were identified as EPCs. In 2003, Hill et al. isolated peripheral blood mononuclear cells (PBMCs) from venous blood by Ficoll gradient centrifugation. To minimize contamination by mature ECs, PBMCs were plated for 48 hours onto a fibronectin plate and then the nonadherent cells were replated onto a fibronectin-coated plate to quantify EPC-derived colonies. Colonies were then characterized by thin, flat cells which radiated from a centre of clustered cells. The assay was commercialized and these colonies were referred to as colony forming unit-Hill (CFU-Hill). The CFU-Hill assay demonstrated an inverse relationship between the circulating CFU-Hill concentration and Framingham cardiovascular risk score in humans [14]. However, later work called into question the reliability of these units for quantifying EPCs and instead noted they may actually represent cells of macrophage/monocyte and/or T-cell origin [45]. Nonetheless, while CFU-based definitions of EPCs may remain imprecise, they have been shown to serve as a marker of clinical endpoints [14].

Identifying the relationship between these two different isolation methods for EPCs is vital to ensuring a uniform definition of EPCs. One study examined the relationship of both methods and revealed that CFU numbers did not correlate to numbers of either CD34+/KDR+ or CD34+/KDR+/CD133+ cells [46]. In 2007, CD34^+^/VEGFR2^+^/CD133^+^ cells were isolated using a cell sorter and assayed using two different endothelial culture systems and hematopoietic colony assays. No endothelial colonies formed and the isolated cells expressed the hematopoietic cell marker, CD45 [44]. One possible explanation for this is that sorted cells may lose critical signaling molecules in the process, which impact the ultimate endothelial phenotype observed in other trials, though this phenomenon is poorly understood. In addition, surface markers used to define EPCs are also found in myeloid-monocytic cells, which may result in isolation of impure EPC populations [47]. Hence, the ideal methodology for isolating and defining an EPC remains the subject of debate, hampering therapeutic advancements.

### 2.2. Sources of EPCs

#### 2.2.1. Bone Marrow

Asahara et al. described the first EPCs as originating in the bone marrow [31]. The bone marrow cells used expressed LacZ (*β*-galactosidase), which was transcriptionally regulated by an endothelial-specific promoter (Tie2 or Flk-1). The group identified LacZ-positive cells in the developing neovasculature in all the models [41]. Since then, several other animal models of ischemia have supported the role of bone marrow-derived mononuclear cells in vascular development [48, 49]. Moreover, several clinical trials have employed bone marrow-derived progenitor cells following revascularization for acute myocardial infarction with varying levels of success in terms of mortality and ventricular recovery [50].

#### 2.2.2. Kidney

Renal progenitor cells (RPCs) were isolated from normal adult human kidney cortical parenchyma with cell-surface markers CD133+ and PAX-2+ (embryonic renal marker). These RPCs were capable of self-replication and could be differentiated in vitro into epithelial or endothelial cells [65]. “Nephrospheres” formed by these cells in culture form the basis of a functional assay used for stem cell isolation. One group utilized the PKH26 tracer to identify CD133+/CD24− cells that differentiated into cells with epithelial, endothelial, or podocytic features [66]. It is thought that these resident RPCs may play a role in renal repair in adults though there is limited data to support this [67, 68].

#### 2.2.3. Blood Vessel Wall

Most models of EPC-mediated vascular repair invoke the homing capability of bone marrow-derived EPCs that are circulating in the blood. It is believed that human aortic endothelial cells (HAECs) or human umbilical vein endothelial cells (HUVECs) from vessel walls are terminally differentiated into mature ECs [69]. However, when HAECs were isolated to examine the hierarchy of ECs based on their proliferative capacity, they were able to be passaged in vitro for >40 population doublings using a single-cell deposition assay, thereby suggesting the existence of resident high proliferative potential endothelial colony forming cells (HPP-ECFCs) line, which may assist in vascular repair [69]. This finding suggested that angiogenesis may in fact be mediated by EPCs [70]. However, increasing evidence suggests that resident ECs actually repair the damaged endothelium, challenging the previous premise that this ability was exclusive to circulating EPCs. Hagensen et al. compared the contribution of circulating and resident cells to vascular repair by transplanting wire-injured carotid artery segments from wild-type mice into Tie2-GFP mice, which expressed GFP in ECs. This model found that the vascular endothelium that formed in response to injury likely arose via migration of surrounding ECs rather than from circulating progenitor cells [71]. Further work is ongoing to identify and characterize the regenerative resident cells within arterial systems.

Recently, in addition to circulating progenitor cells, the existence of resident vascular progenitor cells within the adventitia of blood vessels has been demonstrated. ApoE^−/−^ mice were used to identify adventitial progenitor cells stained by Sca-1, CD34, and Flk-1 [72]. Indeed, Sca-1+ cells were able to differentiate into both ECs and smooth muscle cells (SMCs), thereby potentially contributing to both reendothelialization and/or NI formation, respectively [73]. In 2007, Pasquinelli et al. identified two separate progenitor cell populations between the media and adventitia in human femoral arteries and thoracic aortas [74]. They isolated CD34+ and c-kit+ cells that acquired EC properties when cultured in the presence of VEGF. In 2010, Campagnolo et al. isolated CD34+/CD31− cells from human saphenous veins in CABG patients. These progenitor cells could differentiate into adipocytes, pericytes, and SMCs, stimulated angiogenesis, and improved blood flow when injected into ischemic hind limbs of mice [75].

#### 2.2.4. Adipose Tissue

Adipose tissue was identified as a source of EPCs when Planat-Benard et al. isolated human adipocytes and dedifferentiated them into cells capable of then differentiating into adipocytes or ECs under appropriate conditions [76]. Isolating EPCs from peripheral blood is time consuming whereas adipose tissue may represent an alternative source from which to isolate EPCs. These adipose tissue-derived EPCs (ADEPCs) were capable of enhancing HUVEC capillary-like tube formation on Matrigel [77] and participated in neovascularization when transplanted into rat models of traumatic brain injury [78]. Recent work to characterize the surface markers of ADEPCs has demonstrated the presence of many described EPC markers including CD34, Stro-1, VEGFR-2 (KDR), eNOS, and CD31 [77].

#### 2.2.5. Spleen

Several studies have confirmed an important role for the spleen in EPC mobilization and transplantation [79]. Most hematopoietic stem cells (HSCs) migrate to sinusoids in the bone marrow and spleen and communicate with the endothelium within them, an interaction that seems to be important for their maintenance [80]. Transplantation of spleen-derived mononuclear cells restored endothelium-dependent vasodilation in atherosclerotic ApoE mice, suggesting a splenic source of EPCs for vascular repair [81]. Also, EPCs isolated from spleen homogenate improved reendothelialization and reduced neointima (NI) formation in a model of carotid artery endothelial injury [82].

### 2.3. EPC-Mediated Vascular Repair

#### 2.3.1. Mobilization

The predominance of evidence supports bone marrow-derived EPCs as the most likely source for arterial repair and accordingly we will focus on their mobilization, reviewing various chemokines responsible for guiding EPCs to sites of vascular injury. VEGF, an effective mobilizer and activator of angiogenesis, is believed to induce proliferation, differentiation, and chemotaxis of ECs following vessel injury [83]. Accordingly, augmenting VEGF levels via an adenovirus that expresses VEGF promoted recruitment of EPCs to sites of injury and promoted neovascularization in mice models. This effect is potentially mediated by matrix metallopeptidase-9 (MMP-9) which is converted to the soluble survival factor sKitL, thereby enhancing VEGFR2+ progenitor cells and facilitating transport from osteoblast-rich locations to vascular areas, promoting movement into the circulation [84]. Interestingly, nitric oxide (NO) appears to play a major role in the expression of MMP-9 in its inactive and active form. Studies of endothelial nitric oxide synthase 3 knockout mice (NOS3 KO) have shown reduced expression and activity of MMP-9 even after stimulation with VEGF. These same mice demonstrated reduced neovascularization with induced ischemia and reduced EPC mobilization to ischemic areas [85]. In contrast, AVE9488 (eNOS upregulator) increased NO expression in EPCs and enhanced their migratory capacity [86]. Thus, it appears that NO and MMP-9 play a critical role in facilitating VEGF-mediated angiogenesis.

Secondarily, SDF-1 is a chemokine of growing interest, a constitutively expressed protein that is upregulated by inflammatory mediators, extracellular matrix changes, mechanical forces, and hypoxia [87]. Platelets secrete SDF-1 upon being activated at sites of injury, which may provide the local signal needed to recruit EPCs to the site of injury. In fact, SDF-1 seems to only induce neovascularization in the presence of ischemic injury [88]. In NOS3 KO mice, SDF-1-associated neovascularization and EPC mobilization were silenced, suggesting that VEGF/eNOS signaling pathways likely play important roles in this vascular homeostasis [89]. Additional factors with similar function to SDF-1 include estrogen, statins, and erythropoietin. These factors improve EPC mobilization and neovascularization and inhibit neointima hyperplasia but are similarly silenced in NOS3 KO mice [90–93].

#### 2.3.2. Homing

Understanding the mechanism by which EPCs home to sites of vascular injury is critical to harnessing their therapeutic potential. This process is thought to be similar to the rolling and adhesion behaviour exhibited by leukocytes in the setting of inflammation. EPC homing involves an interaction between EPC surface molecules and associated ligands expressed on dying ECs. P- and E-selectin are believed to play critical roles in this process. Foubert et al. have shed light on this process through their studies on the erythropoietin-producing human hepatocellular carcinoma (Eph) receptor and its associated ephrin ligands, which are regulators of vascular development. The group has shown that Eph4B activation by ephrin-B2-Fc chimeric protein increases the angiogenic potential of human EPCs in a hind limb ischemia mouse model and that this response is blunted with EphB4 siRNA treatment. Eph4B appears to elevate P-selectin glycoprotein ligand-1 expression and EPC adhesion, an effect that can be abrogated with utilizing neutralizing antibodies to E- and P-selectin [94]. *β*2-Integrins on the surface of EPCs mediate migration and adhesion of EPCs to the damaged endothelium as well as neovascularization [95]. High motility group box 1 (HMGB1) has been shown to activate *β*1- and *β*2-integrins on the surface of EPCs by binding to HMGB1 receptors RAGE (receptor for advanced glycation endproducts) and TLR2 (toll-like receptor 2). Interestingly, HMGB1 is released into the extracellular space upon necrosis, but not apoptosis, and was associated with improved homing and adhesion of EPCs to the site of injury [96].

Next, intercellular adhesion molecule-1 (ICAM-1) upregulation in ischemia has been noted to increase EPC recruitment to ischemic limbs [97]. As a hypoxia-responsive gene, integrin-linked kinase (ILK) regulates ICAM-1 expression. Its overexpression is thus associated with increased expression of ICAM-1 as well as SDF-1 in ECs, two key molecules involved in the recruitment of EPCs during vasculogenesis [98]. The synergy between EPC surface proteins and endothelial and/or extracellular ligands is therefore essential for EPC homing to sites of injury (Figure 1).

Apart from EPC migration to sites of vascular injury, EPC invasion of injured tissue is critical for organ repair and function. Gene expression profiling of EPCs and mature ECs has identified the protease Cathepsin L (CathL) as being highly expressed. CathL was shown in vitro to be crucial for matrix degradation and EPC invasion. A CathL-deficient hind limb ischemia mouse model characteristically shows poor limb recovery. As well, when CathL-deficient progenitor cells are infused, impaired homing to sites of injury and impaired neovascularization are observed [99]. EPCs lacking MMP-2 similarly show reduced invasiveness and proliferative capabilities [100]. Hence, numerous molecules have been implicated in the homing of EPCs via a rolling and adhesion model, with additional protease-mediated invasion as required.

#### 2.3.3. Paracrine Effects

It is unlikely that EPC transdifferentiation into mature ECs represents a meaningful reparative mechanism. Rather, an increasing number of studies have shown that these circulating cells may facilitate arterial repair through paracrine influence on neighbouring cells (Figure 1). Many of the cells previously identified as EPCs have more recently been shown to derive from a monocytic lineage, suggesting that they may contribute to vascular repair by releasing paracrine factors such as VEGF [101]. For example, treatment of HUVECs and coronary artery endothelial cells (CAECs) with cultured media (CM) from EPCs or mature ECs demonstrated that EPC-derived CM has higher levels of IL-8 and it demonstrated a stimulatory effect on HUVEC and CAEC proliferation [102].

Paracrine signaling appears to be an important aspect in both early and late EPCs. Early EPCs are derived from a monocytic cell lineage (CD14+) to form spindle-shaped cells after 7 days of culture. In contrast, late EPCs are derived from a CD34+ lineage after 14–28 days in culture and resemble ECs [39]. Early EPCs have a modest contribution to the direct incorporation into the endothelium relative to late EPCs [103] but secrete proangiogenic stimulatory cytokines such as VEGF, fibroblast growth factor (FGF), IL-8, placental growth factor, SDF-1, and platelet derived growth factor (PDGF) [104, 105]. Additionally, the manipulation of the VEGF-Akt pathway could stimulate cell survival and proliferation [106]. These signals are critical as they contribute to endothelial regeneration by recruiting resident endothelial and cardiac progenitor cells to improve endothelial regeneration. Late EPCs have a direct role in neovascularization but a reduced role in paracrine signaling compared to early EPCs. However, they still have been shown to secrete proangiogenic factors including VEGF, IL-8, and PDGF [107]. They suggest that further subsets of EPCs could be defined based on their secretion profile, while modulation of their chemokine profile may help improve their inflammatory profile [108].

More recently, EC-derived microparticles (EMPs) or microvesicles (MV) have been identified as vehicles for different paracrine modes of action. EMPs are complex vesicles shed from activated or apoptotic ECs which play a major role in endothelial function and angiogenesis. EMPs are highly functional molecules with several surface molecules and contain DNA, RNA, or microRNA [109]. These EMPs are taken up by target cells, such as mature ECs, to stimulate endothelial regeneration [47]. Injection of EMPs has been associated with attenuated kidney injury in rats by enhancing proliferation and decreasing apoptosis. This effect was reduced after treating EMPs with RNase or miRNA depletion, implicating a potential function of RNA within EMP for vascular repair [110]. Others have shown that treatment with EMPs accelerates endothelial repair after carotid artery injury in mouse models and that miRNA-126 within EMPs plays a critical role in regulating EC mobilization, proliferation, and regeneration [111]. As we continue to decipher the underlying biology of progenitor cells, the impact of paracrine signaling for vascular repair appears to grow evermore important, presenting both obstacles but also more potential therapeutic targets.

## 3. Therapeutic Potential

Preclinical trials involving EPCs have shown promising results, with EPCs demonstrating the ability to specifically incorporate into the site of injury and mitigate neointima formation [112]. Our group isolated cultured angiogenic cells from patients with CAD and showed that improving CACs could improve reendothelialization [42]. Other substances, including estrogen, G-CSF, and leptin have been demonstrated to mobilize EPCs and enhance arterial repair after vascular injury in animal models [113–115]. Accordingly, endothelial dysfunction is thought to portend cardiovascular disease. Thus, circulating EPC levels may serve as a potential biomarker of endothelial function and integrity, while augmenting EPC levels may represent a viable therapeutic target. Understanding and augmenting endogenous EPCs have attracted considerable attention thus far, so does the transplantation of EPCs directly into sites of injuries. Indeed, these two therapeutic themes represent the majority of applications of EPCs in a therapeutic role to date. However, clinical trials augmenting EPC levels and activity have great variation in EPC isolation techniques and definitions, likely contributing to conflicting results and hindering the advancement of EPCs in the therapeutic realm.

### 3.1. Augmenting Endogenous EPCs

#### 3.1.1. Antihypertensive Therapy

Some common antihypertensive (i.e., blood pressure lowering) agents include angiotensin converting enzyme inhibitors (ACEi), angiotensin II receptor blockers (ARB), and calcium channel blockers (CCB). All of these agents have been extensively studied and yielded an improvement in EPC number and function by differing mechanisms of action. ARBs have improved numbers and function of EPCs by inhibiting oxidative stress [116], while ACEi yielded augmented EPC number and function when given to patients with stable CAD [117]. ACEi have also demonstrated stimulation of nitric oxide activity and diminished oxidative stress in human cells [118]. CCBs such as nifedipine improved EPC function and provided a greater resistance to oxidative stress and apoptosis [119]. Hence, considerable evidence supports the role for antihypertensive agents in improving EPC number and function.

#### 3.1.2. Statin Therapy

Statins were originally developed to modify lipid profiles in patients by reducing LDL and triglyceride levels to improve cardiovascular outcomes. However, clinical studies involving statin therapy in patients with CAD showed higher levels and mobilization of EPCs [120, 121]. These studies showed the initial promise of potentially enhancing endogenous EPCs to improve clinical outcomes. The mechanism behind statin's effect on EPCs is a dose-dependent augmentation of Akt phosphorylation within minutes, which yields an increase in mobilization, migration, proliferation, and survival of EPCs [120]. Elevated EPC levels have also been observed with statin therapy in patients with CAD, acute myocardial infarction, and post-CABG [122–124]. From a vascular repair perspective, patients who develop ISR have also been shown to have functionally impaired EPCs [16, 17]. In a previous review, we demonstrated that statin therapy resulted in a significant rise in circulating EPCs over control, with a median increase of 70.9% (range from 25.8% to 223.5%) [43]. The considerable variance in EPC augmentation following statin therapy most likely reflects differences in the definition of EPCs employed as well as varying isolation techniques.

#### 3.1.3. GSK-3*β*

Glycogen synthase kinase (GSK-3*β*) is a serine/threonine kinase that phosphorylates *β*-catenin to negatively regulate the Wnt signaling pathway [125]. The Wnt signaling pathway plays an essential role in mobilization of EPCs and enhancement of neovascularization [126]. This pathway produces multiple secreted glycoproteins which regulate many cell processes, including hematopoiesis and stem cell function. Our group showed for the first time in an in vitro experiment that the inhibition of GSK-3*β* was associated with increase in EPC levels and reduction of EPC apoptosis. In vivo, we observed that GSK-3*β* inhibition resulted in an improvement in reendothelialization and reduction of neointima formation following injury [42].

Patients with diabetes mellitus (DM) have reduced EPC levels and increased rates of apoptosis. In addition, diabetics have shown higher level of GSK-3*β* activity that resulted in higher levels of phosphorylated *β*-catenin. DM-EPCs are associated with reduced mobilization, migration, and homing to sites of injury [127]. Our group has shown that treatment with GSK-3*β* inhibitors reduced apoptosis, increased VEGF production, and enhanced EPC invasive capacity in vitro. Proteomics analysis of DM-EPC versus normal EPC revealed 37 uniquely regulated proteins. Cathepsin B was identified as the protein that mediates enhanced invasive capacity of EPCs after GSK-3*β* inhibition [128]. Although the direct mechanism of action remains unclear, other studies appear to support our findings. Activation of Wnt/*β*-catenin pathway during human mesenchymal stem cell differentiation was associated with upregulation of Cathepsin B [129]. Although more studies are required on the application of GSK-3*β* inhibition to enhance EPC function, it appears to be a promising avenue for future therapeutic development.

#### 3.1.4. Stents

As discussed, coronary stents have markedly advanced from simple metal scaffolds to sophisticated drug-delivery systems to facilitate arterial healing [5]. Drug-eluting stents (DESs) routinely used for PCI are coated with antiproliferative and anti-inflammatory agents, with first-generation DESs utilizing sirolimus and paclitaxel. Paclitaxel was originally developed as a treatment for ovarian cancer, preventing cellular proliferation via inhibition of microtubule regulation during mitosis [130]. Subsequent work then demonstrated its affinity for inhibiting smooth muscle cell and neointima formation, leading to its development for DESs [131]. Sirolimus (rapamycin) was originally developed as an antifungal agent, but its use was limited due to its immunosuppressive properties. However, it was also noted to suppress NI formation lending itself well to DES implementation. It enacts its effects via blocking G_1_ to S phase progression in cell cycle via inhibition of mammalian target of rapamycin (mTOR) kinase [132]. As the role of EPCs in vascular healing became apparent, novel technologies then focused on discovering means of increasing EPC recruitment and proliferation specifically at the site of stent deployment and subsequent endothelial injury [133].

The first stent to harness the healing capacity of EPCs was the Genous monoclonal anti-human CD34 coated stent [134]. This EPC-capture stent consisted of a stainless steel scaffold covered in a covalently coupled polysaccharide polymer with CD34 antibodies. The Healing-FIM trial compared EPC-capture stent versus bare-metal stents, demonstrating similar NI hyperplasia between the two cohorts and suggesting that the EPC-capture stent failed to inhibit NI hyperplasia [134]. While initially a promising concept, subsequent randomized trials have actually revealed increased ISR with the Genous stent compared to the paclitaxel-eluting stent, paradoxically suggesting increased NI hyperplasia with this technology [135]. These disappointing results are felt to be related to three factors: (i) the stent scaffold itself was an early generation device (thicker struts without any antiproliferative medication), (ii) CD34 is a surface antigen common to various progenitors and may result in nonspecific binding to the stent thereby promoting NI hyperplasia, and (iii) patients with CAD are known to have reduced number and functionality of EPCs [5, 15]. Moreover, the HEALING II registry reported that patients with normal levels of CD34+KDR+ EPC titers had lower rates of ISR compared to patients with reduced levels of EPCs – supporting the notion that augmentation of endogenous EPCs may be key to this success [136]. Hence, attempting to harness rare and dysfunctional cells is likely of little benefit. Accordingly, future trials tried to address the state of the endogenous EPCs available by combining statin therapy with Genous stenting to improve reendothelialization [137]. In a recent 5-year follow-up study of patients treated with Genous stent target lesion revascularization, stent thrombosis, and ISR were stabilized within 12–24 months and up to 5 years [138]. Further data is needed to better understand the role of EPC-capture stent technology in conventional PCI.

To further refine this technology, the EPC-capture stent has been combined with a paclitaxel-coated balloon with intent of reducing the noted NI proliferation and ISR that plagued first-generation Genous stents given its antiproliferative properties. The Perfect Stent study studied 120 patients with CAD who were treated with EPC-capture stent followed by paclitaxel-eluting balloon after dilatation. This approach was successful in reducing restenosis [139] and may well represent the future direction of this technology. Similarly, the Combo stent (OrbusNeich Medical, Fort Lauderdale, USA) is composed of EPC capturing technology combined with a sirolimus-eluting stent to minimize NI formation. The total concentration of sirolimus is half of what is found in a standard DES, but it is released in the same fashion. Preclinical trials in porcine models showed reduced NI thickness in the Combo stent compared to the standard sirolimus-eluting stent, low-dose Combo stent, and everolimus (sirolimus-analog) eluting stent [140]. In 2013, Haude et al. published the prospective, multicenter, randomized evaluation of an abluminal sirolimus coated bioengineered stent (REMEDEE) trial, comparing the Combo stent with the paclitaxel-eluting Taxus Liberte stent and demonstrate that the Combo stent was superior to the paclitaxel stent at 9-month angiographic follow-up because of in-stent late lumen loss with values of 0.39 ± 0.45 mm and 0.44 ± 0.56 mm, respectively [141]. This demonstrated the initial safety and efficacy of Combo stents in comparison to first-generation DESs, but further data is still required to establish this technology. While augmenting and maximizing endogenous EPCs show some promise, an alternative approach involves the direct administration of exogenous EPCs.

### 3.2. Transplantation of Exogenous EPCs

Stem cell therapies have been developed as new treatment regimen for patients suffering from acute myocardial infarctions since the early 2000s. The first of these trials involved the injection of bone marrow-derived mononuclear cells (BMMNC) in 10 patients at the site of infarct following balloon dilatation [142]. Since then, numerous trials injecting BMMNC have been conducted following revascularization by both PCI and CABG to assess myocardial recovery [143–145]. This success has led to large randomized controlled trials of progenitor cell transplantation worldwide [146–148], including myocardial infarction [149].

The procedure involves harvesting bone marrow under general anesthesia from the bony pelvis. CD34+ or CD133+ hematopoietic progenitor cells (similar surface markers as the EPCs defined earlier) are isolated from the BMMNC and then cultured for 2–4 weeks to obtain a sufficient yield of progenitor cells. These cells are then injected directly into the patient's heart at the site of injury during PCI. The timing of stem cell injection varies in studies from first 24–48 hours to four weeks after PCI. Also, the dose of cells that were administered varied considerably within these large trials anywhere from 10^6^ to 10^10^ cells [146–149]. Overall, major trials showed no significant mortality difference between those who received stem cell transplant and those who did not receive cells in the short term. In addition, a Cochrane review on stem cell treatment for acute myocardial infarction revealed no significant reduction in cardiovascular mortality or major adverse cardiovascular events after cell therapy in short- and long-term follow-up [50]. While certainly intriguing and promising, this technology is still in its infancy, and considerable advancements are needed for this to establish itself as a legitimate therapeutic option.

## 4. Conclusion

Our understanding of the complex environment that is the vessel wall remains incomplete. Certainly, the discovery of EPCs holds great potential for both the monitoring and therapy of vascular disease, particularly in repairing the endothelial injury incurred by modern interventions. Be it by augmentation and exploitation of endogenous cells or direct transplantation of cells to areas of need, both approaches show promise. However, despite considerable efforts over the past few decades, our understanding of the definition, signaling, and differentiation of these enigmatic progenitor cells remains imperfect, hampering further developments. Only once these fundamental components are clarified will progenitor cells be able to advance and emerge as a legitimate therapeutic option.

## Figures and Tables

**Table 1:** Evolution of cell-surface markers for endothelial progenitor cell isolation (2014–2016).

		CD34	VEGFR-2/KDR	CD133	CD31	CD45
2014	Liao et al. [51]	Yes	Yes			CD45(dim)
2014	Chi et al. [52]	Yes		Yes		
2015	Shim et al. [53]	Yes			Yes	CD45(dim)
2015	Martí-Fàbregas et al. [54]	Yes	Yes	Yes		
2015	Wang et al. [55]	Yes		Yes		
2015	Tam et al. [56]	Yes	Yes			
2015	Sandra et al. [57]	Yes	Yes	Yes		
2016	Ricottini et al. [58]	Yes	Yes	Yes		CD45(dim)
2016	Lanuti et al. [59]	Yes	Yes	Yes		CD45(dim)
2016	De Ciuceis et al. [60]	Yes	Yes	Yes		
2016	Qin et al. [61]	Yes		Yes		
2016	Niederseer et al., early EPC [62]	Yes				Yes
2016	Niederseer et al., late EPC [62]	Yes	Yes			CD45(dim)
2016	Kung et al. [63]	Yes	Yes	Yes		
2016	Liu et al. [64]	Yes	Yes	Yes		

## References

[1] Manuel D. G., Leung M., Nguyen K., Tanuseputro P., Johansen H. (2003). Burden of cardiovasc disease in Canada. *Canadian Journal of Cardiology*.

[2] Antman E. M., Anbe D. T., Armstrong P. W. (2004). ACC/AHA guidelines for the management of patients with ST-elevation myocardial infarction–executive summary. A report of the American College of Cardiology/American Heart Association Task Force on Practice Guidelines (Writing Committee to revise the 1999 guidelines for the management of patients with acute myocardial infarction). *Journal of the American College of Cardiology*.

[3] Boden W. E., O'Rourke R. A., Teo K. K. (2007). Optimal medical therapy with or without PCI for stable coronary disease. *New England Journal of Medicine*.

[4] Froeschl M., Olsen S., Ma X., O'Brien E. R. (2004). Current understanding of in-stent restenosis and the potential benefit of drug eluting stents. *Current Drug Targets—Cardiovascular and Haematological Disorders*.

[5] Simard T., Hibbert B., Ramirez F. D., Froeschl M., Chen Y.-X., O'Brien E. R. (2014). The evolution of coronary stents: a brief review. *Canadian Journal of Cardiology*.

[6] Bønaa K. H., Mannsverk J., Wiseth R. (2016). Drug-eluting or bare-metal stents for coronary artery disease. *New England Journal of Medicine*.

[7] Mauri L., Hsieh W.-H., Massaro J. M., Ho K. K. L., D'Agostino R., Cutlip D. E. (2007). Stent thrombosis in randomized clinical trials of drug-eluting stents. *New England Journal of Medicine*.

[8] Farb A., Sangiorgi G., Carter A. J. (1999). Pathology of acute and chronic coronary stenting in humans. *Circulation*.

[9] Dzau V. J., Gnecchi M., Pachori A. S., Morello F., Melo L. G. (2005). Therapeutic potential of endothelial progenitor cells in cardiovascular diseases. *Hypertension*.

[10] Mak K.-H., Belli G., Ellis S. G., Moliterno D. J. (1996). Subacute stent thrombosis: evolving issues and current concepts. *Journal of the American College of Cardiology*.

[11] Muniyappa R., Sowers J. R. (2014). Glycogen synthase kinase-3*β* and cathepsin B in diabetic endothelial progenitor cell dysfunction: an old player finds a new partner. *Diabetes*.

[12] Wilcox J. N., Okamoto E.-I., Nakahara K.-I., Vinten-Johansen J. (2001). Perivascular responses after angioplasty which may contribute to postangioplasty restenosis. *Annals of the New York Academy of Sciences*.

[13] Asahara T., Takahashi T., Masuda H. (1999). VEGF contributes to postnatal neovascularization by mobilizing bone marrow-derived endothelial progenitor cells. *EMBO Journal*.

[14] Hill J. M., Zalos G., Halcox J. P. J. (2003). Circulating endothelial progenitor cells, vascular function, and cardiovascular risk. *New England Journal of Medicine*.

[15] Werner N., Kosiol S., Schiegl T. (2005). Circulating endothelial progenitor cells and cardiovascular outcomes. *New England Journal of Medicine*.

[16] George J., Herz I., Goldstein E. (2003). Number and adhesive properties of circulating endothelial progenitor cells in patients with in-stent restenosis. *Arteriosclerosis, Thrombosis, and Vascular Biology*.

[17] Hibbert B., Chen Y.-X., O'Brien E. R. (2004). c-kit-immunopositive vascular progenitor cells populate human coronary in-stent restenosis but not primary atherosclerotic lesions. *American Journal of Physiology—Heart and Circulatory Physiology*.

[18] Schächinger V., Erbs S., Elsässer A. (2006). Improved clinical outcome after intracoronary administration of bone-marrow-derived progenitor cells in acute myocardial infarction: final 1-year results of the REPAIR-AMI trial. *European Heart Journal*.

[19] Werner N., Junk S., Laufs U. (2003). Intravenous transfusion of endothelial progenitor cells reduces neointima formation after vascular injury. *Circulation research*.

[20] George J., Goldstein E., Abashidze S. (2004). Circulating endothelial progenitor cells in patients with unstable angina: association with systemic inflammation. *European Heart Journal*.

[21] Massa M., Rosti V., Ferrario M. (2005). Increased circulating hematopoietic and endothelial progenitor cells in the early phase of acute myocardial infarction. *Blood*.

[22] Padfield G. J., Newby D. E., Mills N. L. (2010). Understanding the role of endothelial progenitor cells in percutaneous coronary intervention. *Journal of the American College of Cardiology*.

[23] Florey H. W., Greer S. J., Poole J. C., Werthessen N. T. (1961). The pseudointima lining fabric grafts of the aorta. *British Journal of Experimental Pathology*.

[24] Jordan G. L., Stump M. M., de Bakey M. E., Halpert B. (1962). Endothelial lining of dacron prostheses of porcine thoracic aortas. *Proceedings of the Society for Experimental Biology and Medicine*.

[25] Stump M. M., Jordan G. L., De Bakey M. E., Halpert B. (1962). The endothelial lining of homografts and dacron prostheses in the canine aorta. *The American Journal of Pathology*.

[26] Stump M. M., Jordan G. L., Debakey M. E., Halpert B. (1963). Endothelium grown from circulating blood on isolated intravascular. *The American journal of pathology*.

[27] Feigl W., Susani M., Ulrich W., Matejka M., Losert U., Sinzinger H. (1985). Organisation of experimental thrombosis by blood cells—evidence of the transformation of mononuclear cells into myofibroblasts and endothelial cells. *Virchows Archiv A Pathological Anatomy and Histopathology*.

[28] Pasquinelli G., Preda P., Curti T., D'Addato M., Laschi R. (1987). Endothelialization of a new Dacron graft in an experimental model: light microscopy, electron microscopy and immunocytochemistry. *Scanning Microscopy*.

[29] Campbell J. H., Efendy J. L., Campbell G. R. (1999). Novel vascular graft grown within recipient's own peritoneal cavity. *Circulation Research*.

[30] Hibbert B., Simard T., O'Brien E. R., Allan D. S., Strunk D. (2012). Endothelial progenitors and repair of cardiovascular disease. *Regenerative Therapy Using Blood-Derived Stem Cells*.

[31] Asahara T., Murohara T., Sullivan A. (1997). Isolation of putative progenitor endothelial cells for angiogenesis. *Science*.

[32] Hibbert B., Ma X., Pourdjabbar A. (2011). Pre-procedural Atorvastatin mobilizes endothelial progenitor cells: clues to the salutary effects of statins on healing of stented human arteries. *PLOS ONE*.

[33] Janssens S., Dubois C., Bogaert J. (2006). Autologous bone marrow-derived stem-cell transfer in patients with ST-segment elevation myocardial infarction: double-blind, randomised controlled trial. *The Lancet*.

[34] Ma X., Hibbert B., Dhaliwal B. (2010). Delayed re-endothelialization with rapamycin-coated stents is rescued by the addition of a glycogen synthase kinase-3 inhibitor. *Cardiovascular Research*.

[35] Takahashi T., Kalka C., Masuda H. (1999). Ischemia- and cytokine-induced mobilization of bone marrow-derived endothelial progenitor cells for neovascularization. *Nature Medicine*.

[36] Jackson K. A., Majka S. M., Wang H. (2001). Regeneration of ischemic cardiac muscle and vascular endothelium by adult stem cells. *Journal of Clinical Investigation*.

[37] Asahara T., Masuda H., Takahashi T. (1999). Bone marrow origin of endothelial progenitor cells responsible for postnatal vasculogenesis in physiological and pathological neovascularization. *Circulation Research*.

[38] Lyden D., Hattori K., Dias S. (2001). Impaired recruitment of bone-marrow-derived endothelial and hematopoietic precursor cells blocks tumor angiogenesis and growth. *Nature Medicine*.

[39] Xu S., Zhu J., Yu L., Fu G. (2012). Endothelial progenitor cells: current development of their paracrine factors in cardiovascular therapy. *Journal of Cardiovascular Pharmacology*.

[40] Timmermans F., Plum J., Yöder M. C., Ingram D. A., Vandekerckhove B., Case J. (2009). Endothelial progenitor cells: identity defined?. *Journal of Cellular and Molecular Medicine*.

[41] Sirker A. A., Astroulakis Z. M. J., Hill J. M. (2009). Vascular progenitor cells and translational research: the role of endothelial and smooth muscle progenitor cells in endogenous arterial remodelling in the adult. *Clinical Science*.

[42] Hibbert B., Ma X., Pourdjabbar A. (2009). Inhibition of endothelial progenitor cell glycogen synthase kinase-3*β* results in attenuated neointima formation and enhanced re-endothelialization after arterial injury. *Cardiovascular Research*.

[43] Hibbert B., Simard T., Ramirez F. D. (2013). The effect of statins on circulating endothelial progenitor cells in humans: a systematic review. *Journal of Cardiovascular Pharmacology*.

[44] Case J., Mead L. E., Bessler W. K. (2007). Human CD34+AC133+VEGFR-2+ cells are not endothelial progenitor cells but distinct, primitive hematopoietic progenitors. *Experimental Hematology*.

[45] Kovacic J. C., Boehm M. (2009). Resident vascular progenitor cells: an emerging role for non-terminally differentiated vessel-resident cells in vascular biology. *Stem Cell Research*.

[46] George J., Shmilovich H., Deutsch V., Miller H., Keren G., Roth A. (2006). Comparative analysis of methods for assessment of circulating endothelial progenitor cells. *Tissue Engineering*.

[47] Zhang M., Malik A. B., Rehman J. (2014). Endothelial progenitor cells and vascular repair. *Current Opinion in Hematology*.

[48] Kawamoto A., Gwon H.-C., Iwaguro H. (2001). Therapeutic potential of ex vivo expanded endothelial progenitor cells for myocardial ischemia. *Circulation*.

[49] Kocher A. A., Schuster M. D., Szabolcs M. J. (2001). Neovascularization of ischemic myocardium by human bone-marrow-derived angioblasts prevents cardiomyocyte apoptosis, reduces remodeling and improves cardiac function. *Nature Medicine*.

[50] Fisher S. A., Zhang H., Doree C., Mathur A., Martin-Rendon E. (2015). Stem cell treatment for acute myocardial infarction. *The Cochrane Database of Systematic Reviews*.

[51] Liao Y.-F., Feng Y., Chen L.-L., Zeng T.-S., Yu F., Hu L.-J. (2014). Coronary heart disease risk equivalence in diabetes and arterial diseases characterized by endothelial function and endothelial progenitor cell. *Journal of Diabetes and Its Complications*.

[52] Chi J., Hong X., Wang Y., Zhao J., Yang W. (2014). Inverse correlation between circulating endothelial progenitor cells with CD34+CD133+ and the severity of coronary atherosclerosis assessed by syntax score. *American Journal of the Medical Sciences*.

[53] Shim Y., Nam M. H., Hyuk S. W., Yoon S. Y., Song J. M. (2015). Concurrent hypermulticolor monitoring of CD31, CD34, CD45 and CD146 endothelial progenitor cell markers for acute myocardial infarction. *Analytica Chimica Acta*.

[54] Martí-Fàbregas J., Delgado-Mederos R., Crespo J. (2015). Circulating endothelial progenitor cells and the risk of vascular events after ischemic stroke. *PLOS ONE*.

[55] Wang L., Wang X., Su H. (2015). Recombinant human erythropoietin improves the neurofunctional recovery of rats following traumatic brain injury via an increase in circulating endothelial progenitor cells. *Translational Stroke Research*.

[56] Tam J. C. W., Ko C. H., Lau K. M. (2015). Enumeration and functional investigation of endothelial progenitor cells in neovascularization of diabetic foot ulcer rats with a Chinese 2-herb formula. *Journal of Diabetes*.

[57] Sandra F., Oktaviono Y. H., Widodo M. A., Dirgantara Y., Chouw A., Sargowo D. (2015). Endothelial progenitor cells proliferated via MEK-dependent p42 MAPK signaling pathway. *Molecular and Cellular Biochemistry*.

[58] Ricottini E., Madonna R., Grieco D. (2016). Effect of high-dose atorvastatin reload on the release of endothelial progenitor cells in patients on long-term statin treatment who underwent percutaneous coronary intervention (from the ARMYDA-EPC Study). *American Journal of Cardiology*.

[59] Lanuti P., Rotta G., Almici C. (2016). Endothelial progenitor cells, defined by the simultaneous surface expression of VEGFR2 and CD133, are not detectable in healthy peripheral and cord blood. *Cytometry Part A*.

[60] De Ciuceis C., Rossini C., Tincani A. (2016). Effect of antihypertensive treatment with lercanidipine on endothelial progenitor cells and inflammation in patients with mild to moderate essential hypertension. *Blood Pressure*.

[61] Qin G., Chen Y., Li H. (2016). Melittin inhibits tumor angiogenesis modulated by endothelial progenitor cells associated with the SDF-1*α*/CXCR4 signaling pathway in a UMR-106 osteosarcoma xenograft mouse model. *Molecular Medicine Reports*.

[62] Niederseer D., Steidle-Kloc E., Mayr M. (2016). Effects of a 12-week alpine skiing intervention on endothelial progenitor cells, peripheral arterial tone and endothelial biomarkers in the elderly. *International Journal of Cardiology*.

[63] Kung C.-T., Su C.-M., Chen C. T. (2016). Circulating endothelial progenitor cells may predict outcomes in adult patients with severe sepsis in the emergency department. *Clinica Chimica Acta*.

[64] Liu J.-H., Chen Y., Zhen Z. (2016). Relation between endothelial progenitor cells and arterial stiffness in patients with psoriasis. *Journal of Dermatology*.

[65] Bussolati B., Bruno S., Grange C. (2005). Isolation of renal progenitor cells from adult human kidney. *American Journal of Pathology*.

[66] Bombelli S., Zipeto M. A., Torsello B. (2013). PKHhigh cells within clonal human nephrospheres provide a purified adult renal stem cell population. *Stem Cell Research*.

[67] Bussolati B., Camussi G. (2015). Therapeutic use of human renal progenitor cells for kidney regeneration. *Nature Reviews Nephrology*.

[68] Goligorsky M. S., Yasuda K., Ratliff B. (2010). Dysfunctional endothelial progenitor cells in chronic kidney disease. *Journal of the American Society of Nephrology*.

[69] Ingram D. A., Mead L. E., Moore D. B., Woodard W., Fenoglio A., Yoder M. C. (2005). Vessel wall-derived endothelial cells rapidly proliferate because they contain a complete hierarchy of endothelial progenitor cells. *Blood*.

[70] Kovacic J. C., Moore J., Herbert A., Ma D., Boehm M., Graham R. M. (2008). Endothelial progenitor cells, angioblasts, and angiogenesis—old terms reconsidered from a current perspective. *Trends in Cardiovascular Medicine*.

[71] Hagensen M. K., Raarup M. K., Mortensen M. B. (2012). Circulating endothelial progenitor cells do not contribute to regeneration of endothelium after murine arterial injury. *Cardiovascular Research*.

[72] Hu Y., Zhang Z., Torsney E. (2004). Abundant progenitor cells in the adventitia contribute to atheroscleroses of vein grafts in ApoE-deficient mice. *Journal of Clinical Investigation*.

[73] Torsney E., Hu Y., Xu Q. (2005). Adventitial progenitor cells contribute to arteriosclerosis. *Trends in Cardiovascular Medicine*.

[74] Pasquinelli G., Tazzari P. L., Vaselli C. (2007). Thoracic aortas from multiorgan donors are suitable for obtaining resident angiogenic mesenchymal stromal cells. *Stem Cells*.

[75] Campagnolo P., Cesselli D., Al Haj Zen A. (2010). Human adult vena saphena contains perivascular progenitor cells endowed with clonogenic and proangiogenic potential. *Circulation*.

[76] Planat-Benard V., Silvestre J.-S., Cousin B. (2004). Plasticity of human adipose lineage cells toward endothelial cells: physiological and therapeutic perspectives. *Circulation*.

[77] Zhou L., Xia J., Qiu X. (2015). In vitro evaluation of endothelial progenitor cells from adipose tissue as potential angiogenic cell sources for bladder angiogenesis. *PLoS ONE*.

[78] Xue S., Zhang H.-T., Zhang P. (2010). Functional endothelial progenitor cells derived from adipose tissue show beneficial effect on cell therapy of traumatic brain injury. *Neuroscience Letters*.

[79] Zhao X., Wu N., Huang L. (2010). Endothelial progenitor cells and spleen: new insights in regeneration medicine. *Cytotherapy*.

[80] Kiel M. J., Yilmaz Ö. H., Iwashita T., Yilmaz O. H., Terhorst C., Morrison S. J. (2005). SLAM family receptors distinguish hematopoietic stem and progenitor cells and reveal endothelial niches for stem cells. *Cell*.

[81] Wassmann S., Werner N., Czech T., Nickenig G. (2006). Improvement of endothelial function by systemic transfusion of vascular progenitor cells. *Circulation Research*.

[82] Werner L., Deutsch V., Barshack I., Miller H., Keren G., George J. (2005). Transfer of endothelial progenitor cells improves myocardial performance in rats with dilated cardiomyopathy induced following experimental myocarditis. *Journal of Molecular and Cellular Cardiology*.

[83] Asahara T., Takahashi T., Masuda H. (1999). VEGF contributes to postnatal neovascularization by mobilizing bone marrow-derived endothelial progenitor cells. *The EMBO Journal*.

[84] Heissig B., Hattori K., Dias S. (2002). Recruitment of stem and progenitor cells from the bone marrow niche requires MMP-9 mediated release of Kit-ligand. *Cell*.

[85] Aicher A., Heeschen C., Mildner-Rihm C. (2003). Essential role of endothelial nitric oxide synthase for mobilization of stem and progenitor cells. *Nature Medicine*.

[86] Sasaki K.-I., Heeschen C., Aicher A. (2006). *Ex vivo* pretreatment of bone marrow mononuclear cells with endothelial NO synthase enhancer AVE9488 enhances their functional activity for cell therapy. *Proceedings of the National Academy of Sciences of the United States of America*.

[87] Ceradini D. J., Kulkarni A. R., Callaghan M. J. (2004). Progenitor cell trafficking is regulated by hypoxic gradients through HIF-1 induction of SDF-1. *Nature Medicine*.

[88] Massberg S., Konrad I., Schürzinger K. (2006). Platelets secrete stromal cell-derived factor 1*α* and recruit bone marrow-derived progenitor cells to arterial thrombi in vivo. *Journal of Experimental Medicine*.

[89] Hiasa K.-I., Ishibashi M., Ohtani K. (2004). Gene transfer of stromal cell-derived factor-1*α* enhances ischemic vasculogenesis and angiogenesis via vascular endothelial growth factor/endothelial nitric oxide synthase-related pathway: next-generation chemokine therapy for therapeutic neovascularization. *Circulation*.

[90] Urao N., Okigaki M., Yamada H. (2006). Erythropoietin-mobilized endothelial progenitors enhance reendothelialization via Akt-endothelial nitric oxide synthase activation and prevent neointimal hyperplasia. *Circulation Research*.

[91] Walter D. H., Rittig K., Bahlmann F. H. (2002). Statin therapy accelerates reendothelialization: a novel effect involving mobilization and incorporation of bone marrow-derived endothelial progenitor cells. *Circulation*.

[92] Laufs U., Werner N., Link A. (2004). Physical training increases endothelial progenitor cells, inhibits neointima formation, and enhances angiogenesis. *Circulation*.

[93] D'Uscio L. V., Smith L. A., Santhanam A. V., Richardson D., Nath K. A., Katusic Z. S. (2007). Essential role of endothelial nitric oxide synthase in vascular effects of erythropoietin. *Hypertension*.

[94] Foubert P., Silvestre J.-S., Souttou B. (2007). PSGL-1-mediated activation of EphB4 increases the proangiogenic potential of endothelial progenitor cells. *Journal of Clinical Investigation*.

[95] Chavakis E., Aicher A., Heeschen C. (2005). Role of *β*2-integrins for homing and neovascularization capacity of endothelial progenitor cells. *Journal of Experimental Medicine*.

[96] Chavakis E., Hain A., Vinci M. (2007). High-mobility group box 1 activates integrin-dependent homing of endothelial progenitor cells. *Circulation Research*.

[97] Yoon C.-H., Hur J., Oh I.-Y. (2006). Intercellular adhesion molecule-1 is upregulated in ischemic muscle, which mediates trafficking of endothelial progenitor cells. *Arteriosclerosis, Thrombosis, and Vascular Biology*.

[98] Lee S.-P., Youn S.-W., Cho H.-J. (2006). Integrin-linked kinase, a hypoxia-responsive molecule, controls postnatal vasculogenesis by recruitment of endothelial progenitor cells to ischemic tissue. *Circulation*.

[99] Urbich C., Heeschen C., Aicher A. (2005). Cathepsin L is required for endothelial progenitor cell-induced neovascularization. *Nature Medicine*.

[100] Cheng X. W., Kuzuya M., Nakamura K. (2007). Mechanisms underlying the impairment of ischemia-induced neovascularization in matrix metalloproteinase 2-deficient mice. *Circulation Research*.

[101] Rehman J., Li J., Orschell C. M., March K. L. (2003). Peripheral blood “endothelial progenitor cells” are derived from monocyte/macrophages and secrete angiogenic growth factors. *Circulation*.

[102] He T., Peterson T. E., Katusic Z. S. (2005). Paracrine mitogenic effect of human endothelial progenitor cells: role of interleukin-8. *American Journal of Physiology—Heart and Circulatory Physiology*.

[103] Fadini G. P., Baesso I., Albiero M., Sartore S., Agostini C., Avogaro A. (2008). Technical notes on endothelial progenitor cells: ways to escape from the knowledge plateau. *Atherosclerosis*.

[104] Yang Z., von Ballmoos M. W., Faessler D. (2010). Paracrine factors secreted by endothelial progenitor cells prevent oxidative stress-induced apoptosis of mature endothelial cells. *Atherosclerosis*.

[105] Wyler von Ballmoos M., Yang Z., Völzmann J., Baumgartner I., Kalka C., Di Santo S. (2010). Endothelial progenitor cells induce a phenotype shift in differentiated endothelial cells towards PDGF/PDGFR*β* axis-mediated angiogenesis. *PLoS ONE*.

[106] Meng F., Liu L., Chin P. C., D'Mello S. R. (2002). Akt is a downstream target of NF-*κ*B. *The Journal of Biological Chemistry*.

[107] Kebir A., Harhouri K., Guillet B. (2010). CD146 short isoform increases the proangiogenic potential of endothelial progenitor cells in vitro and in vivo. *Circulation Research*.

[108] Zhang Y., Ingram D. A., Murphy M. P. (2009). Release of proinflammatory mediators and expression of proinflammatory adhesion molecules by endothelial progenitor cells. *American Journal of Physiology—Heart and Circulatory Physiology*.

[109] Dignat-George F., Boulanger C. M. (2011). The many faces of endothelial microparticles. *Arteriosclerosis, Thrombosis, and Vascular Biology*.

[110] Cantaluppi V., Gatti S., Medica D. (2012). Microvesicles derived from endothelial progenitor cells protect the kidney from ischemia-reperfusion injury by microRNA-dependent reprogramming of resident renal cells. *Kidney International*.

[111] Jansen F., Yang X., Hoelscher M. (2013). Endothelial microparticle-mediated transfer of microRNA-126 promotes vascular endothelial cell repair via spred1 and is abrogated in glucose-damaged endothelial microparticles. *Circulation*.

[112] Werner N., Priller J., Laufs U. (2002). Bone marrow-derived progenitor cells modulate vascular reendothelialization and neointimal formation: effect of 3-hydroxy-3-methylglutaryl coenzyme a reductase inhibition. *Arteriosclerosis, Thrombosis, and Vascular Biology*.

[113] Kong D., Melo L. G., Gnecchi M. (2004). Cytokine-induced mobilization of circulating endothelial progenitor cells enhances repair of injured arteries. *Circulation*.

[114] Schroeter M. R., Leifheit M., Sudholt P. (2008). Leptin enhances the recruitment of endothelial progenitor cells into neointimal lesions after vascular injury by promoting integrin-mediated adhesion. *Circulation Research*.

[115] Strehlow K., Werner N., Berweiler J. (2003). Estrogen increases bone marrow-derived endothelial progenitor cell production and diminishes neointima formation. *Circulation*.

[116] Yoshida Y., Fukuda N., Maeshima A. (2011). Treatment with valsartan stimulates endothelial progenitor cells and renal label-retaining cells in hypertensive rats. *Journal of Hypertension*.

[117] Cacciatore F., Bruzzese G., Vitale D. F. (2011). Effects of ACE inhibition on circulating endothelial progenitor cells, vascular damage, and oxidative stress in hypertensive patients. *European Journal of Clinical Pharmacology*.

[118] Jacoby D. S., Rader D. J. (2003). Renin-angiotensin system and atherothrombotic disease: from genes to treatment. *Archives of Internal Medicine*.

[119] Sugiura T., Kondo T., Kureishi-Bando Y. (2008). Nifedipine improves endothelial function: role of endothelial progenitor cells. *Hypertension*.

[120] Llevadot J., Murasawa S., Kureishi Y. (2001). HMG-CoA reductase inhibitor mobilizes bone marrow-derived endothelial progenitor cells. *The Journal of Clinical Investigation*.

[121] Vasa M., Fichtlscherer S., Adler K. (2001). Increase in circulating endothelial progenitor cells by statin therapy in patients with stable coronary artery disease. *Circulation*.

[122] Leone A. M., Rutella S., Giannico M. B. (2008). Effect of intensive vs standard statin therapy on endothelial progenitor cells and left ventricular function in patients with acute myocardial infarction: Statins for regeneration after acute myocardial infarction and PCI (STRAP) trial. *International Journal of Cardiology*.

[123] Schmidt-Lucke C., Fichtlscherer S., Aicher A. (2010). Quantification of circulating endothelial progenitor cells using the modified ISHAGE protocol. *PLoS ONE*.

[124] Spadaccio C., Pollari F., Casacalenda A. (2010). Atorvastatin increases the number of endothelial progenitor cells after cardiac surgery: a randomized control study. *Journal of Cardiovascular Pharmacology*.

[125] Yost C., Torres M., Miller J. R., Huang E., Kimelman D., Moon R. T. (1996). The axis-inducing activity, stability, and subcellular distribution of *β*-catenin is regulated in Xenopus embryos by glycogen synthase kinase 3. *Genes and Development*.

[126] Aicher A., Kollet O., Heeschen C. (2008). The wnt antagonist dickkopf-1 mobilizes vasculogenic progenitor cells via activation of the bone marrow endosteal stem cell niche. *Circulation Research*.

[127] Jarajapu Y. P. R., Grant M. B. (2010). The promise of cell-based therapies for diabetic complications challenges and solutions. *Circulation Research*.

[128] Hibbert B., Lavoie J. R., Ma X. (2014). Glycogen synthase kinase-3*β* inhibition augments diabetic endothelial progenitor cell abundance and functionality via cathepsin B: a novel therapeutic opportunity for arterial repair. *Diabetes*.

[129] Herencia C., Martínez-Moreno J. M., Herrera C. (2012). Nuclear translocation of *β*-catenin during mesenchymal stem cells differentiation into hepatocytes is associated with a tumoral phenotype. *PLoS ONE*.

[130] Jordan M. A., Toso R. J., Thrower D., Wilson L. (1993). Mechanism of mitotic block and inhibition of cell proliferation by taxol at low concentrations. *Proceedings of the National Academy of Sciences of the United States of America*.

[131] Axel D. I., Kunert W., Göggelmann C. (1997). Paclitaxel inhibits arterial smooth muscle cell proliferation and migration in vitro and in vivo using local drug delivery. *Circulation*.

[132] Gallo R., Padurean A., Jayaraman T. (1999). Inhibition of intimal thickening after balloon angioplasty in porcine coronary arteries by targeting regulators of the cell cycle. *Circulation*.

[133] Padfield G. J., Tura O., Haeck M. L. A. (2010). Circulating endothelial progenitor cells are not affected by acute systemic inflammation. *American Journal of Physiology—Heart and Circulatory Physiology*.

[134] Aoki J., Serruys P. W., van Beusekom H. (2005). Endothelial progenitor cell capture by stents coated with antibody against CD34: the HEALING-FIM (Healthy Endothelial Accelerated Lining Inhibits Neointimal Growth-First in Man) registry. *Journal of the American College of Cardiology*.

[135] Beijk M. A. M., Klomp M., Verouden N. J. W. (2010). Genous™ endothelial progenitor cell capturing stent vs. the Taxus Liberté stent in patients with de novo coronary lesions with a high-risk of coronary restenosis: a randomized, single-centre, pilot study. *European Heart Journal*.

[136] Duckers H. J., Silber S., de Winter R. (2007). Circulating endothelial progenitor cells predict angiographic and intravascular ultrasound outcome following percutaneous coronary interventions in the HEALING-II trial: evaluation of an endothelial progenitor cell capturing stent. *EuroIntervention*.

[137] den Dekker W. K., Houtgraaf J. H., Rowland S. M. (2014). Efficiency of statin treatment on EPC recruitment depends on baseline EPC titer and does not improve angiographic outcome in coronary artery disease patients treated with the Genous stent. *Cell Transplant*.

[138] Pereira-da-Silva T., Bernardes L., Cacela D. (2013). Safety and effectiveness of the genous endothelial progenitor cell-capture stent: follow-up to 5 years. *Journal of Invasive Cardiology*.

[139] Wöhrle J., Birkemeyer R., Markovic S. (2011). Prospective randomised trial evaluating a paclitaxel-coated balloon in patients treated with endothelial progenitor cell capturing stents for de novo coronary artery disease. *Heart*.

[140] Granada J. F., Inami S., Aboodi M. S. (2010). Development of a novel prohealing stent designed to deliver sirolimus from a biodegradable abluminal matrix. *Circulation: Cardiovascular Interventions*.

[141] Haude M., Lee S. W. L., Worthley S. G. (2013). The REMEDEE trial: a randomized comparison of a combination sirolimus-eluting endothelial progenitor cell capture stent with a paclitaxel-eluting stent. *JACC: Cardiovascular Interventions*.

[142] Strauer B. E., Brehm M., Zeus T. (2002). Repair of infarcted myocardium by autologous intracoronary mononuclear bone marrow cell transplantation in humans. *Circulation*.

[143] Assmus B., Schächinger V., Teupe C. (2002). Transplantation of progenitor cells and regeneration enhancement in acute myocardial infarction (TOPCARE-AMI). *Circulation*.

[144] Meyer G. P., Wollert K. C., Drexler H. (2006). Stem cell therapy: a new perspective in the treatment of patients with acute myocardial infarction. *European Journal of Medical Research*.

[145] Stamm C., Westphal B., Kleine H.-D. (2003). Autologous bone-marrow stem-cell transplantation for myocardial regeneration. *The Lancet*.

[146] Schächinger V., Erbs S., Elsässer A. (2006). Intracoronary bone marrow-derived progenitor cells in acute myocardial infarction. *New England Journal of Medicine*.

[147] Traverse J. H., Henry T. D., Pepine C. J. (2012). Effect of the use and timing of bone marrow mononuclear cell delivery on left ventricular function after acute myocardial infarction: the TIME randomized trial. *The Journal of the American Medical Association*.

[148] Sürder D., Manka R., Lo Cicero V. (2013). Intracoronary injection of bone marrow-derived mononuclear cells early or late after acute myocardial infarction: effects on global left ventricular function. *Circulation*.

[149] Gao L. R., Pei X. T., Ding Q. A. (2013). A critical challenge: dosage-related efficacy and acute complication intracoronary injection of autologous bone marrow mesenchymal stem cells in acute myocardial infarction. *International Journal of Cardiology*.

